# Should the microbiota of raw milk cheeses play a role in the definition of geographical indications and quality schemes within the European Union?

**DOI:** 10.1128/msystems.00520-23

**Published:** 2023-11-02

**Authors:** Pier Sandro Cocconcelli, Monica Gatti, Giorgio Giraffa, Marco Gobbetti, Rosalba Lanciotti, Lorenzo Morelli, Erasmo Neviani, Eugenio Parente

**Affiliations:** 1 Università Cattolica del Sacro Cuore, Piacenza-Cremona, Italy; 2 University Parma, Parma, Italy; 3 CREA-Lodi, Lodi, Italy; 4 Free University Bolzano, Bolzano, Italy; 5 Alma Mater University Bologna, Bologna, Italy; 6 University Basilicata, Potenza, Italy; Teagasc Food Research Centre, Fermoy, Cork, Ireland

**Keywords:** raw milk cheese, microbiota, metagenomics

## LETTER

This letter is to share with the readers of *mSystems* our criticisms about work by Fontana et al. which has been recently published (1).

Based on a rather simplistic sampling approach, the authors conclude that their results “highlighted the need for a profound rethinking of the current PDO designation with a focus on the production site-specific microbial metabolism to understand and guarantee the organoleptic features of the final product recognized as PDO.” We feel that this conclusion is not supported by the data and that the experimental approach and the interpretation of results indicate a lack of familiarity with the complex relationship between cheese making technologies, microbial successions, and product properties in raw milk cheeses. Above all, it highlights the lack of knowledge of how a PDO product is linked to the production area and specific technology. We also feel that the literature cited is definitely biased: many authoritative and highly cited papers on the structure and dynamics of microbial communities in raw milk PDO cheeses have not been cited neither in the introduction nor in the discussion, providing a lopsided picture of the scientific literature. This can be easily proven by carrying out a simple search combined with a citation overview on Scopus with the very limited set of keywords provided in the paper and comparing the results with the references cited by Fontana et al. (210 more than the six mentioned in the paper which are neither the most recent nor the most significant).

Our many criticisms, however, are related to the sampling approach. Raw milk PDO cheeses (not only Italian ones) are extremely variable, not only among producers but also, above all, throughout ripening. Neither of these factors is taken into account in the sampling approach.

Starting from a merely descriptive, albeit based on state-of-the-art metagenomic approaches, photograph of the microbial community of the products, the authors suggest the need for a “profound rethinking” of one of the main quality branding systems for traditional products in the European Union. A much more comprehensive sampling coverage, combined with longitudinal studies of the microbiota, would have barely provided enough data to support this statement.

The paper has further flaws:

- assuming that the analysis of the metagenome of a product at the time of consumption can characterize the microbiota and its evolution during ripening is wrong (2)
- the resolution of metagenomic approaches is being greatly exaggerated: it is unclear how long and short reads were combined, and the information presented on Metagenome-Assembeld-Genome quality is limited and fails to comply with the minimum standards for accountability (3)
- use of parametric tests in key statistical comparisons for microbiome analysis, without correction for multiple comparisons, is, simply put, wrong (4)
- the authors overlook the importance of technological aspect of dairy production and their effect as drivers of microbial succession (5–13)
- the authors seem to ignore the foundation of the quality scheme in the EU. PDO is linked to the production area, raw materials, and technology: microorganisms and fermentation processes are not the only drivers of product quality and identity (7)

We are all too aware of the difficulties of the peer review process but we cannot help being surprised that the reviewers did not notice the points we have raised.

## EDITOR'S NOTE

*Ed. Note: The author of the published article did not feel that a response was necessary.*
